# SalivaDirect: an alternative to a conventional RNA extraction protocol for molecular detection of SARS-CoV-2 in a clinical setting

**DOI:** 10.1128/spectrum.03272-23

**Published:** 2023-11-28

**Authors:** Mohammad Khaja Mafij Uddin, Mohammad Enayet Hossain, Jenifar Quaiyum Ami, Rashedul Hasan, Md. Mahmudul Hasan, Ashabul Islam, Md. Jahid Hasan, Nusrat Jahan Shaly, Shahriar Ahmed, Pushpita Samina, Mohammed Ziaur Rahman, Mustafizur Rahman, Sayera Banu

**Affiliations:** 1 Infectious Diseases Division, International Centre for Diarrhoeal Disease Research, Dhaka, Bangladesh; 2 Nutrition and Clinical Services Division, International Centre for Diarrhoeal Disease Research, Dhaka, Bangladesh; 3 Centre for Health Economics and Policy Analysis, McMaster University, Hamilton, Ontario, Canada; Foundation for Innovative New Diagnostics, Geneve, Switzerland

**Keywords:** SARS-CoV-2, SalivaDirect, Nasopharyngeal swab, Saliva, COVID-19, RT-qPCR

## Abstract

Severe acute respiratory syndrome coronavirus 2 (SARS-CoV2) continues to spread and evolve, giving rise to new surges in cases and deaths. Although nasopharyngeal swab (NPS) samples are the gold standard for diagnosing SARS-CoV2, NPS sampling is invasive and requires skilled staff, specialized transport medium, and nucleic acid extraction. The SalivaDirect method uses saliva samples and a simplified, flexible approach to overcome these drawbacks. In our study conducted at the COVID-19 Screening Unit of the Dhaka Hospital of icddr,b (International Centre for Diarrheal Disease Research, Bangladesh), the diagnostic performance of SalivaDirect and NPS was compared using paired samples. SARS-CoV2 was detected by performing RT-qPCR using RNA extracted from saliva samples with the SalivaDirect method and from the NPS with extracted RNA. A total of 200 participants were enrolled from February to March 2021, among whom 78 (39.0%) tested positive for SAR-CoV2 from at least one sample. Among the 78 participants testing positive, 65 (83.3%) tested positive in both specimens, eight (10.3%) tested positive only in NPS, and five (6.4%) tested positive only in saliva. SalivaDirect had a sensitivity of 89.0% and specificity of 96.1%, with NPS as the reference test. The sensitivity of NPS and SalivaDirect was 93.6% and 89.7% respectively, with a composite reference standard (where patients are defined as positive if tested positive in either method) as the reference. Our findings demonstrated that the SalivaDirect method can be used as an alternative to NPS in our clinical setting and also supports the use of SalivaDirect in other settings.

**IMPORTANCE:**

Affordable and accessible tests for COVID-19 allow for timely disease treatment and pandemic management. SalivaDirect is a faster and easier method to implement than NPS sampling. Patients can self-collect saliva samples at home or in other non-clinical settings without the help of a healthcare professional. Sample processing in SalivaDirect is less complex and more adaptable than in conventional nucleic acid extraction methods. We found that SalivaDirect has good diagnostic performance and is ideal for large-scale testing in settings where supplies may be limited or trained healthcare professionals are unavailable.

## INTRODUCTION

Severe acute respiratory syndrome coronavirus 2 (SARS-CoV-2) is the pathogen that caused the coronavirus disease 2019 (COVID-19) pandemic (1). The first confirmed COVID-19 case was found in December 2019 in Wuhan, China. It became a global pandemic, affecting 126,359,540 people globally and triggering 2,769,473 deaths during the study period as of 28th March 2021 (2). As SARS-CoV-2 continues to mutate and spread, countries need to build on their current pandemic management to stop new variants of SARS-CoV-2 from causing surges in cases and deaths by enhancing diagnosis capabilities. There are various methods to test for SARS-CoV-2, among which quantitative reverse transcription PCR (RT-qPCR) test using nasopharyngeal swab (NPS) sampling is the gold standard. The NPS became the sample of choice for the test based on its performance in detecting other respiratory diseases (3). However, obtaining NPS has its limitations. The sample collection process is invasive and causes patient discomfort, leading to reluctance toward repeated sampling. Sampling may also induce coughing and sneezing, which puts healthcare workers at risk of nosocomial infection (4). Using saliva as the sample for RT-qPCR is a feasible alternative, and it has several advantages over NPS. Saliva sampling is non-invasive, can be self-administered by patients, and has flexible transportation requirements (5). This increases patient compliance, reduces pressure on healthcare workers, and facilitates more accessible sample transport. Using saliva instead of NPS has already been proven to be a possible alternative to NPS in many studies (6 – 10).

In our previous study, we performed RT-qPCR using RNA extracted from saliva (6). Nucleic acid extraction is a time-consuming and expensive process, which has become costlier due to the global shortages in supply since the beginning of the pandemic. The processing time, cost, and accessibility of COVID-19 testing can be further improved using the SalivaDirect protocol developed at the Yale School of Public Health. SalivaDirect replaces RNA extraction with an enzymatic step and a 5 minutes heating step, which are faster and simpler (11). The protocol is adaptable with a wide combination of equipment and reagents. It does not require a specific transport medium, allowing labs to modify the protocol according to the material available to the setting. SalivaDirect received Emergency Use Authorization (EUA) on August 15, 2020 (12).

In this cross-sectional study, we aimed to investigate the feasibility and use of the SalivaDirect protocol. We compared the results with those of the gold standard NPS method for the detection of SARS-CoV-2 among suspected patients and their contacts visiting the dedicated COVID-19 Screening Unit of Dhaka Hospital of icddr,b (International Centre for Diarrheal Disease Research, Bangladesh).

## MATERIALS AND METHODS

### Study setting

This study was conducted between February and March 2021 at the COVID-19 Screening Unit of the Dhaka Hospital of icddr,b. Suspected patients with symptoms of COVID-19 and those in contact with confirmed COVID-19 cases, with or without the symptoms of COVID-19, were enrolled during the study period. Patients gave informed written consent, or where written consent was not possible, assent was obtained from each participant before enrollment. Demographic and clinical data, including signs and symptoms of COVID-19, hospitalization, and traveling history, were collected from all participants. Participants were excluded if they did not consent, were unable to provide the required (1.0–2.0 mL) amount of specimens, or if they were less than 10 years old. The Research Review Committee and the Ethical Review Committee of icddr,b’s Institutional Review Board approved the study protocol.

### Sample collection

Paired samples of the NPS and saliva were collected from 200 individuals. NPS samples were collected by a trained nurse following appropriate aseptic conditions. Each patient was asked to tilt their head back; the swab was inserted horizontally into the nasopharynx until resistance was met, rotated up to five times, and left in place for 5–10 seconds to absorb the secretions. The swab was placed in 1.0 mL viral transport medium (VTM), and the handle was broken off before sealing the VTM (13). Saliva samples were self-collected by patients under the supervision of a healthcare worker who ensured appropriate collection volume and quality. The patients were asked to refrain from eating, drinking, using mouthwash, or brushing their teeth in the morning prior to saliva collection. Patients placed a flocked swab under their tongues for 2–3 minutes to pool saliva in their mouth and drool saliva into a sterile container until 1.0–2.0 mL of saliva was collected. The swab was discarded after use, and no transportation medium was used. Patients were advised to wash their hands before and after the procedure. The collected paired samples were stored temporarily in a cool box (2–8°C) before being transported to the laboratory for testing within 3 hours of collection. The laboratory technicians were blinded to the patient details and results of the other method.

### Viral RNA processing and RT-qPCR assay

All the specimens were processed for SARS-CoV-2 detection at the Virology Laboratory of the Institute of Epidemiology Disease Control and Research (IEDCR) and icddr,b. One hundred forty microliter of the NPS was used to extract viral RNA using the QIAamp Viral RNA Mini Kit (QIAGEN, Germany). A portion (5 µL) of the resuspended RNA was used for the RT-qPCR assay.

The SalivaDirect protocol followed the method described by Vogels et al. (11). Briefly, 2.5 µL (50 mg/mL) of Proteinase K was added to 50 µL of saliva in 8-strip PCR tubes. The tubes were placed in a rack and vortexed for 1 minute at 3,200 rpm. The samples were then heated for 5 minutes at 95°C in a thermocycler, and then 5 µL of the processed saliva was used as a template for the RT-qPCR assay.

Following the extraction procedure for NPS and SalivaDirect protocol, an RT-qPCR assay was performed using the Reliance One-Step Multiplex SARS-CoV-2 RT-PCR Supermix Kit (Bio-Rad). The amplification assay comprises the 2019-nCoV_N1 (N1) and the human RNase P control (RP) primer-probe sets developed by US CDC. The RT-qPCR master mix was prepared following the manufacturer’s recommended instructions, with 400 nM of N1 forward and reverse primer, 200 nM of the N1 probe, 150 nM of the RP forward and reverse primer, and 200 nM of the RP probe per reaction. Thermocycler conditions were as follows: 10 minutes at 52°C, 2 minutes at 95°C, and 45 cycles of 10 seconds at 95°C and 30 seconds at 55°C on the CFX96 Touch Real-Time PCR System (Bio-Rad Laboratories Inc. USA). A sample was considered positive for SARS-CoV-2 when the Ct value for N1 was ≤37 and considered negative when the Ct value was ≥37.

### Statistical analysis

Data analysis was performed using the statistical software STATA/SE version 17. Descriptive statistics were presented as mean ± standard deviation for continuous variables and percentages for categorical variables. GraphPad Prism 9.0 was used for generating figures. A paired *t*-test was used to determine the statistically significance of the mean difference between Ct values. Receiver operating characteristic (ROC) curve analysis was performed to compare the sensitivity and specificity of SalivaDirect with those of NPS. The consistency of the results obtained by the SalivaDirect and the NPS methods was evaluated using inter-observer reliability analysis with the Kappa statistic. For each of the tests, *P* < 0.05 was considered statistically significant. This study’s composite reference standard (CRS) consisted of the RT-qPCR results with NPS and SalivaDirect test that determined the final diagnosis of COVID-19.

## RESULTS

Two hundred paired NPS and saliva specimens were collected from suspected COVID-19 patients who either showed signs or symptoms of COVID-19 or were in contact with confirmed COVID-19 cases. Among the study participants, 116 (58%) were male, and the median age (interquartile range [IQR]) was 18 (12 to 71) years. Among the participants, 183 were symptomatic for COVID-19, while 17 participants were asymptomatic. A total of 78 (39.0%) patients were confirmed as COVID-19 positive by RT-qPCR from at least one specimen type (NPS or saliva). The positivity rates with RT-qPCR using the regular RNA extraction procedure from NPS and SalivaDirect process from saliva were 36.5% (73/200) and 35% (70/200), respectively (Table 1). Among the 78 positive specimens, 65 (83.3%) were found to be positive in both sampling methods, 8 (10.3%) samples were found positive in NPS alone, and 5 (6.4%) were found positive in the SalivaDirect method alone.

**TABLE 1:** Determination of diagnostic performances of SalivaDirect with NPS RT-qPCR as the reference standard

Test method	NPS								
SalivaDirect	Positive	Negative	Total	Sensitivity (95% CI)	Specificity (95% CI)	PPV (95% CI)	NPV (95% CI)	Agreement (%)	Cohen κ
Positive	65	5	70	89.0 (79.5–95.1)	96.1 (91.1–98.7)	60.7 (48.3 to 72.2)	99.2 (95.8 to 99.9)	93.5	0.86
Negative	8	122	130						
Total	73	127	200						

RT-qPCR results of NPS were used as a reference standard to determine the diagnostic performance of the SalivaDirect protocol. The sensitivity and specificity of the RT-qPCR with the SalivaDirect method were 89.0% (95% CI, 79.5% to 95.1%) and 96.1% (95% CI, 91.1% to 98.7%), respectively. The positive predictive (PPV) value and negative predictive values (NPV) were 60.7% (95% CI, 48.3% to 72.2 %) and 99.2 (95% CI, 95.8% to 99.9%), respectively. The study results revealed 93.5% agreement (k coefficient, 0.86; *P* <0.001) between these two methods in detecting SARS-CoV-2 viral RNA in the specimens.

The individual diagnostic performances of NPS and SalivaDirect were also calculated compared to that of the composite reference standard (CRS), where patients are considered positive if they test positive through either NPS and SalivaDirect or both (Table 2). The sensitivity of NPS and SalivaDirect was 93.6% (95% CI, 85.7% to 97.9%) and 89.7% (95% CI, 80.79% to 95.47%), respectively. The specificity and PPV were 100% for both methods, whereas the NPV was 99.30% (95% CI, 95.91 to 99.99) and 99.56% (95% CI, 96.30 to 100.00) for SalivaDirect and NPS, respectively. The agreement with the CRS was 97.5% and 96.0% for NPS and SalivaDirect, respectively.

**TABLE 2:** Diagnostic validity parameters of NPS or SalivaDirect compared to those of the CRS (positive in both or either test)

Comparison with CRS (positive = 78)								
Test method	Positive	Negative	Sensitivity (95% CI)	Specificity (95% CI)	PPV (95% CI)	NPV (95% CI)	Agreement (%)	Cohen κ
NPS	73	5	93.6 (85.7 to 97.9)	100 (97.0 to 100)	100	99.6% (96.3 to 100)	97.5	0.95
SalivaDirect	70	8	89.7% (80.8 to 95.5)	100 (97.0 to 100)	100	99.3% (95.91 to 99.9)	96.0	0.91

**Fig 1:**
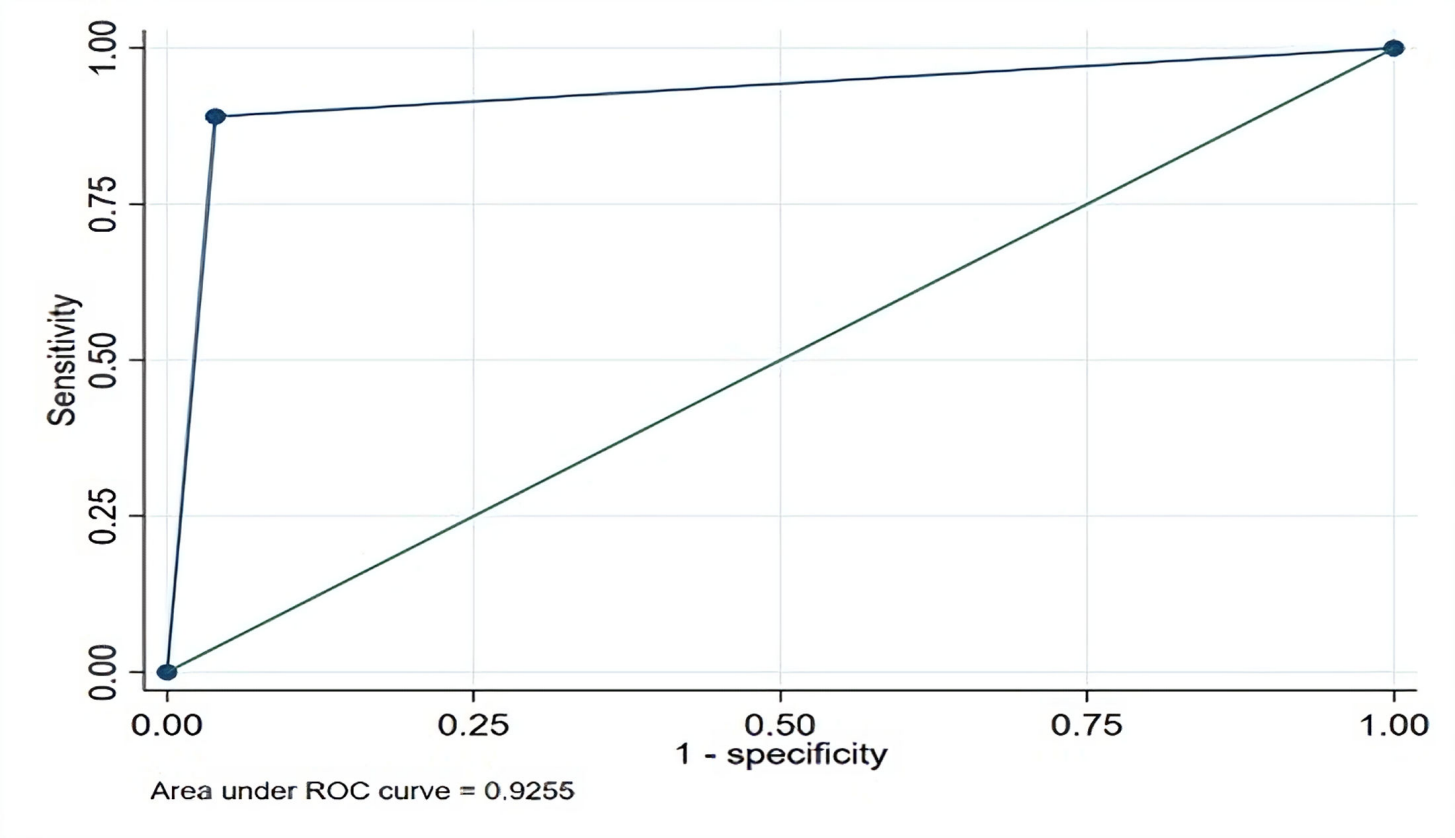
ROC curve analysis of the SalivaDirect procedure when compared to NPS

The diagnostic performance of SalivaDirect was also compared to that of NPS based on the Ct values derived from RT-qPCR (Table 3). The sensitivity and specificities of SalivaDirect with NPS as the reference were found to be highest (100%) when NPS had Ct values of <20 in RT-qPCR. The sensitivity for the SalivaDirect method was 88.5% and 50% when the Ct values for NPS were 20–30 and >30, respectively. Additionally, the ROC curve was used to evaluate the diagnostic performance of the SalivaDirect compared to NPS. The area under the ROC curve was 0.925, showing a 92.5% probability that SalivaDirect will correctly distinguish a positive patient from a negative one based on the NPS results (Fig. 1).

**TABLE 3:** Detection with SalivaDirect according to different ranges of ct values with NPS

Ct values with NPS	Detection with SalivaDirect
<20	37/37 (100%)
20–30	23/26 (88.5%)
>30	5/10 (50%)

**Fig 2:**
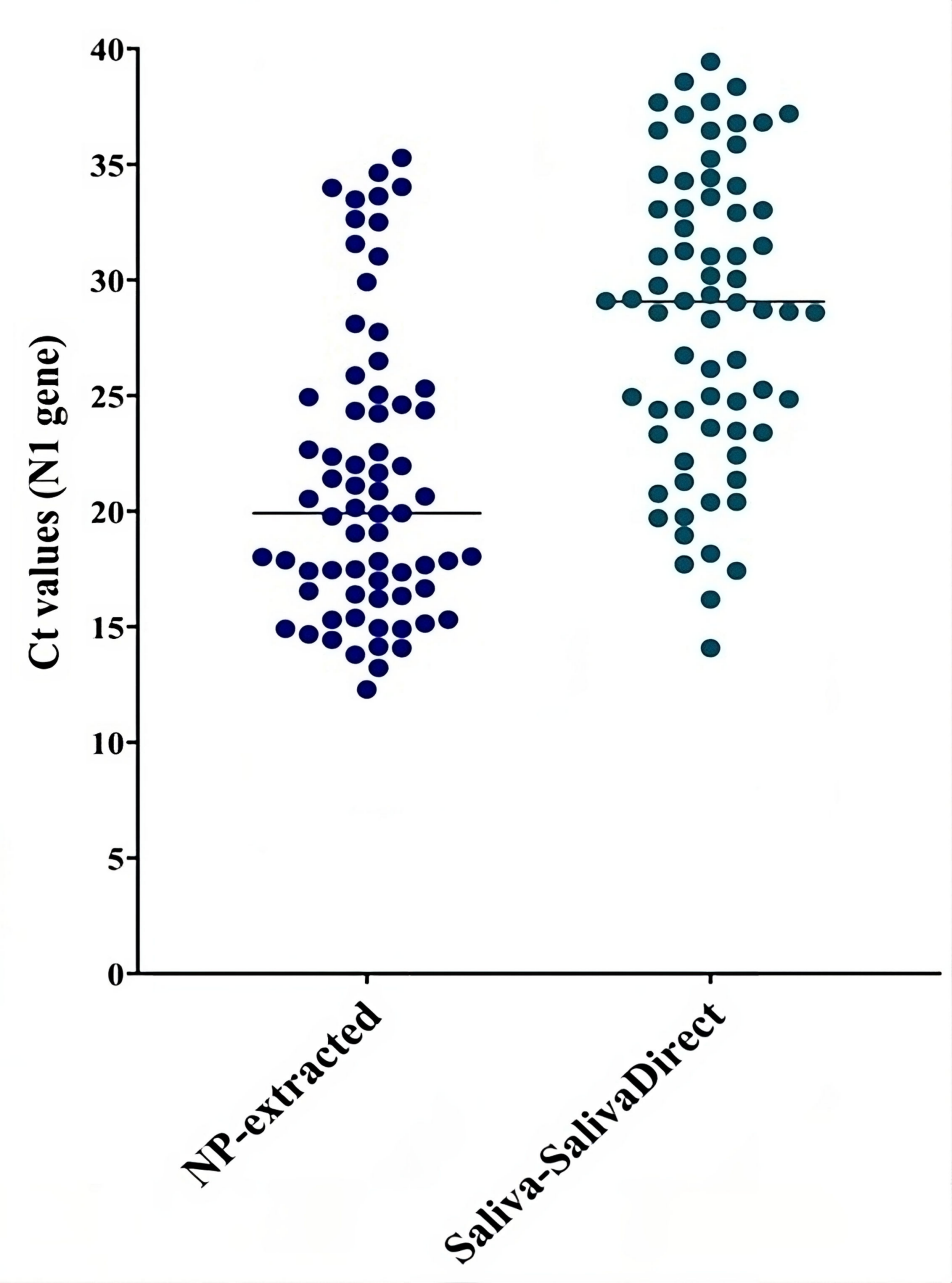
Comparison of ct values of N1 gene target in paired nasopharyngeal (extracted) and saliva (SalivaDirect) specimens

**Fig 3:**
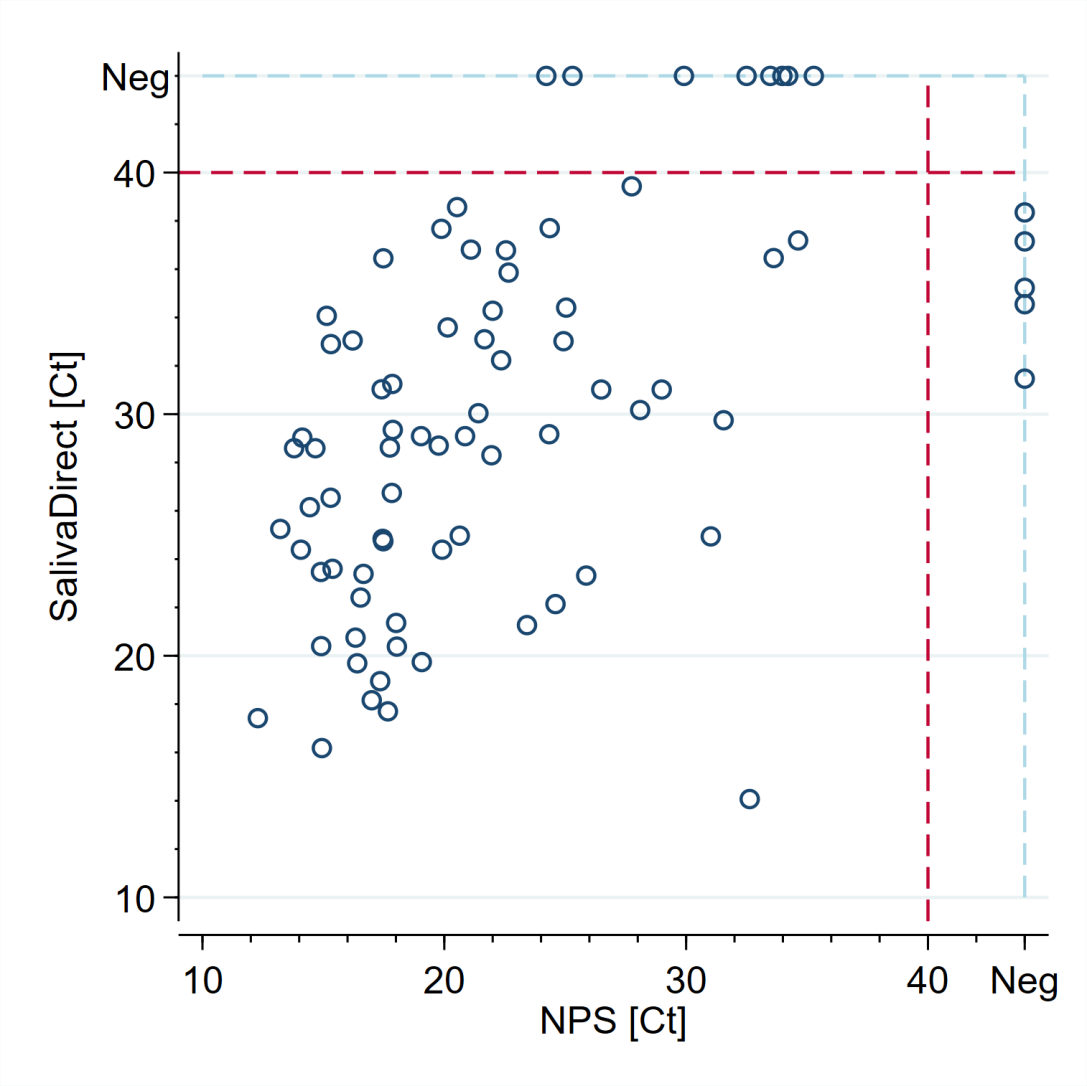
Comparison of SalivaDirect and NPS ct values among the positive specimens

A paired *t*-test revealed a significant difference (*P* < 0.001) in the median Ct values for the N1 gene between the two methods. The NPS method had a median Ct value of 19.8, with an IQR of 12.29 to 34.63. In contrast, the SalivaDirect method had a higher median Ct value of 29.06, with an IQR of 14.07 to 39.43 (Fig. 2). Discordant results were found in 13 pairs of specimens: SARS-CoV2 was detected only via NPS in eight pairs and only from saliva in five pairs (Fig. 3).

## DISCUSSION

Collecting the optimal sample for detecting SARS-CoV-2 is vital to ending the COVID-19 pandemic. As salivary glands contain the ACE-2 receptor, SARS-CoV-2 can directly infect salivary glands and epithelial cells in the mouth or enter saliva from the mucociliary escalator, respiratory tract, nasal cavity, blood, or liquid droplets, making saliva a suitable specimen for detecting the presence of SARS-CoV-2 (14 – 16). Using saliva instead of NPS may increase patient compliance and reduce the chance of nosocomial infection, as patients might be induced to sneeze or cough during uncomfortable NPS collection.

In our testing, with NPS as the reference test, SalivaDirect had a sensitivity of 89.0%, a specificity of 96.1, and a PPV of 60.7 (95% CI, 48.3 to 72.2). In a study conducted by Tee et al., where they also used crude saliva without transportation media, the sensitivity of SalivaDirect was found to be 91.3%, with NPS as the reference (17). Another study reported 88.2% sensitivity when comparing the SalivaDirect protocol with the reference standard NPS (18). A separate study reported a sensitivity of 94.3% for SalivaDirect compared to matching NPS samples among a combined cohort of symptomatic and asymptomatic individuals, similar to our study (19). Our study findings are consistent with those of these studies and the first report of the SalivaDirect method by Vogels et al. where the sensitivity was found to be 89.5%. Vogels et al. validated multiple kits and equipment and found that the Reliance One-Step Multiplex RT-qPCR Supermix, which we used, was among the kits showing higher sensitivity. In our previous study, RT-qPCR was performed with RNA extracted from saliva, and the results were compared to those of the conventional NPS. The sensitivity was 80.3%, which is lower than that observed in the current study (6).

We must consider that the primers in the RT-qPCR are highly specific to COVID-19 and a false positive result is only possible if samples are mishandled. Comparing the SalivaDirect approach with NPS as the reference standard would bias NPS and falsely lower the PPV for SalivaDirect; therefore, a CRS that defines patients as positive if found positive in either method will be useful (17). In the current study, when NPS and SalivaDirect RT-qPCR were individually compared to the CRS, we found closer sensitivities of 89.7% and 93.6% for SalivaDirect and NPS, respectively. Both methods have missed positive cases that the other method detected—five cases missed by NPS and eight cases missed by SalivaDirect. All 13 discrepant sample pairs were from symptomatic, unvaccinated individuals. The five cases, detected only by SalivaDirect, involved individuals aged 20–54 years, three of whom came into contact with a confirmed COVID-19 patient within 14 days prior to enrollment. The remaining eight cases, detected only by NPS, involved individuals aged 20–52 years, four of whom had contact with confirmed COVID-19 patients. In the missed cases, the Ct values in RT-qPCR for the positive paired samples were >20, which indicates lower viral loads. Moreover, the median Ct value for SalivaDirect was lower than the median Ct value of NPS overall (19.8 vs 29.06), meaning lower viral load in the collected saliva than in the NPS, which may be due to higher viral loads in the NPS or the liquid volume of saliva contributing to the dilution of viral content (17). Both samples were collected at the same stage of disease onset. The stage of the disease can influence the locality of the virus replication as it is found in the oral mucosa during disease onset and travels down the respiratory tract as the disease progresses (20). The variation in diagnosis could therefore be caused by the stage of disease rather than the quality of the collected sample. Another study reported a similar sensitivity (88.1%) for SalivaDirect compared to the CRS consisting of RT-qPCR from NPS, Saliva-extracted, and SalivaDirect (17).

Based on the convenience in sampling, processing, and evaluating the performance of SalivaDirect in diagnosing COVID-19, we recommend the use of RT-qPCR from SalivaDirect in place of NPS in Bangladesh and in settings where NPS sampling is not possible due to shortages of healthcare workers, unavailability of nucleic acid extraction techniques, or patient incompliance. It can also be suggested for mass surveillance as the slight loss in sensitivity is offset by the significant boost in sample collection and processing efficiency. Due to the flexible nature of the protocol, the transportation RT-qPCR kit and thermal cycler used have an effect on the lower limit of detection, and choosing the optimal combination, when possible, will increase the sensitivity. Using both methods simultaneously can also be considered for epidemiology studies. Low- and middle-income countries can benefit from SalivaDirect as it is cheaper due to not requiring swabs, VTMs, and extraction. The pandemic caused a shortage of laboratory supplies and equipment, making it harder to carry out nucleic acid extractions with commercial kits and generic reagents (21). Having a protocol that can be adapted to available laboratory supplies will help avoid dependence on any particular supplier in future emergencies.

This study had some limitations. We did not consider the stage of disease during the sample collection or follow-up with patients later. A follow-up sample could have helped us further understand the effect of disease onset on viral temporality and speciality. Some saliva samples had mucus and inadequate volume despite a healthcare worker present during sample collection. As self-collection of samples might increase the rate of such occurrences, a subset of samples could have been collected without supervision to evaluate this possibility. This study was conducted before the emergence of the Omicron variant. Omicron and similar emerging variants should have a higher detection rate through SalivaDirect than through NPS due to having different tropism and a higher detection rate in saliva (22, 23). Studies must address these concerns to help us better prepare for future outbreaks and reemergence of respiratory diseases such as COVID-19.
